# A small step towards an important goal: fragment screen of the c-di-AMP-synthesizing enzyme CdaA

**DOI:** 10.1107/S205979832400336X

**Published:** 2024-04-29

**Authors:** Piotr Neumann, Jana L. Heidemann, Jan Wollenhaupt, Achim Dickmanns, Michael Agthe, Manfred S. Weiss, Ralf Ficner

**Affiliations:** a Department of Molecular Structural Biology, Institute of Microbiology and Genetics, GZMB, Georg-August-University Göttingen, Justus-von-Liebig-Weg 11, 37077 Göttingen, Germany; b Macromolecular Crystallography, Helmholtz-Zentrum Berlin, Albert-Einstein-Strasse 15, 12489 Berlin, Germany; c Institut für Nanostruktur- und Festkörperphysik, Universität Hamburg, Luruper Chaussee 149, 22761 Hamburg, Germany; d Cluster of Excellence ‘Multiscale Bioimaging: From Molecular Machines to Networks of Excitable Cells’ (MBExC), University of Göttingen, 37075 Göttingen, Germany; McGill University, Canada

**Keywords:** crystallographic fragment screening, CdaA, *Listeria monocytogenes*, cyclic di-AMP, drug design

## Abstract

CdaA is the most widespread diadenylate cyclase in many bacterial species, including several multidrug-resistant human pathogens. The enzymatic product of CdaA, cyclic di-AMP, is a secondary messenger that is essential for the viability of many bacteria. Its absence in humans makes CdaA a very promising and attractive target for the development of new antibiotics. Here, the structural results are presented of a crystallographic fragment screen against CdaA from *Listeria monocytogenes*, a saprophytic Gram-positive bacterium and an opportunistic food-borne pathogen that can cause listeriosis in humans and animals. Two of the eight fragment molecules reported here were localized in the highly conserved ATP-binding site. These fragments could serve as potential starting points for the development of antibiotics against several CdaA-dependent bacterial species.

## Abstract 2

Crystallographic fragment screening of the bacterial target *Listeria monocytogenes* CdaA is reported.

## 1. Introduction

**Figure 1:**
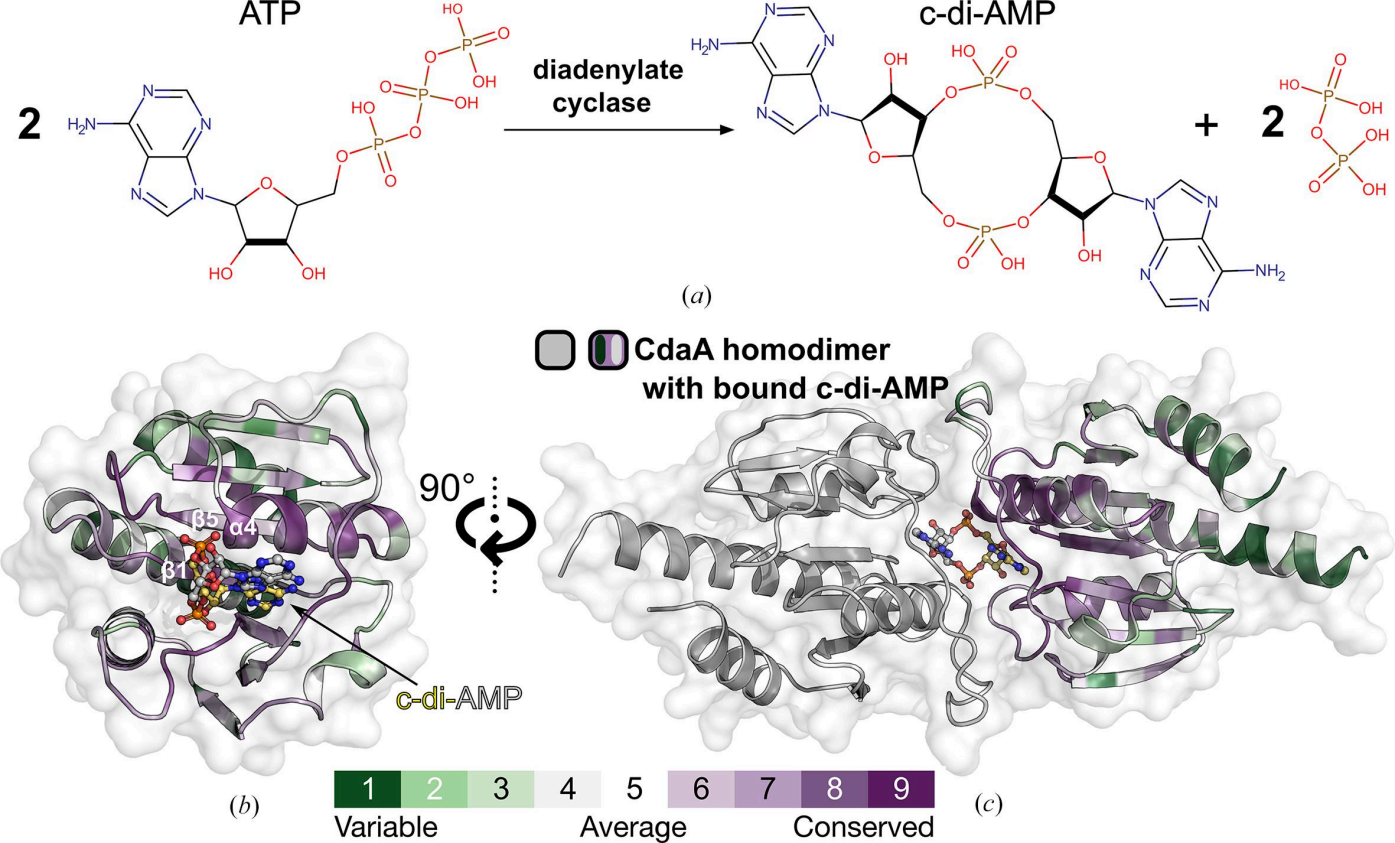
Crystal structure of *L. monocytogenes* Δ100CdaA. (*a*) Biochemical reaction of DAC. (*b*) Overall structure of the truncated monomeric Δ100CdaA. The c-di-AMP molecule, depicted in ball-and-stick mode, marks the active site. Color coding is according to conservation score: green, low; white, medium; purple, high. (*c*) Cartoon representation of the catalytically active Δ100CdaA dimer in its postcatalytic state with c-di-AMP bound in the active site (PDB entry 6hvl).

Multidrug-resistant bacteria constitute a major global health threat in modern healthcare (Cassini *et al.*, 2019 ▸). Recent reports from the World Health Organization (WHO) indicate that antimicrobial resistance contributes to approximately 700 000 deaths globally each year, with concerns about a potential significant rise in fatalities without effective intervention (an estimated prediction of ten million deaths annually by 2050; Asokan *et al.*, 2019 ▸). The misuse and overuse of antibiotics in recent decades have fueled the relentless evolution of bacterial pathogens, severely limiting our effective antimicrobial arsenal. Consequently, there is an urgent need for innovative antibacterial strategies to combat this escalating global health crisis. In this context, the bacterial cyclic di-adenosine monophosphate (c-di-AMP) signaling pathway, which is not present in humans (Rosenberg *et al.*, 2015 ▸), has emerged as a promising antibacterial target (Corrigan & Gründling, 2013 ▸; Römling, 2008 ▸; Schuster *et al.*, 2016 ▸). Functioning as a secondary messenger, c-di-AMP intricately regulates essential bacterial cellular processes, including osmolyte and potassium ion homeostasis, biofilm formation, cell-wall integrity, DNA integrity scanning, modulation of metabolism, virulence and stress response (Gundlach *et al.*, 2016 ▸; Mehne *et al.*, 2013 ▸; Rørvik *et al.*, 2020 ▸; Whiteley *et al.*, 2017 ▸). Diadenylate cyclases (DACs), which are c-di-AMP-synthesizing enzymes, contribute significantly to this intricate signaling network by catalysing the conversion of two adenosine triphosphate (ATP) molecules into one c-di-AMP molecule (Fig. 1 ▸) in a metal-ion-dependent manner (Witte *et al.*, 2008 ▸). Hence, DACs are essential in the bacterial cell. Remarkably, they exhibit extensive structural conservation across diverse species (mainly Gram-positive bacteria), highlighting the profound importance of DACs and rendering them attractive candidates for drug design.

Five different classes of DACs are known to date: CdaA, DisA, CdaS, CdaM and CdaZ (Blötz *et al.*, 2017 ▸; Commichau *et al.*, 2019 ▸; Corrigan & Gründling, 2013 ▸; Römling, 2008 ▸), of which the first three have been structurally characterized. They all share the highly conserved DAC domain (Fig. 1 ▸) accompanied by regulatory domains (Commichau *et al.*, 2019 ▸). The DAC domain of CdaA reveals an overall globular fold, with a central seven-stranded mixed β-sheet flanked by five α-helices. The well conserved active site is located between α-helix 4, β-strand 1 and β-strand 5 as well as several loops connecting secondary-structure elements (Fig. 1 ▸). The synthesis of c-di-AMP requires the transient dimerization of two ATP-loaded CdaA molecules in a face-to-face orientation, as seen in the hexagonal CdaA–c-di-AMP complex structure in the postcatalytic state (PDB entry 6hvl; Fig. 1 ▸; Heidemann *et al.*, 2019 ▸; Rosenberg *et al.*, 2015 ▸; Witte *et al.*, 2008 ▸). Most bacteria that are known to synthesize c-di-AMP possess only one class of DAC: either DisA or CdaA. The latter has been described to be the most prevalent DAC domain-containing protein in several bacterial species (Commichau *et al.*, 2019 ▸), including human pathogens such as *Listeria monocytogenes*, *Borrelia turicatea*, *Enterococcus faecalis*, *Staphylococcus aureus*, *Mycobacterium tuberculosis* and *Streptococcus pneumoniae* (Bai *et al.*, 2012 ▸; Corrigan *et al.*, 2011 ▸; Jackson-Litteken *et al.*, 2021 ▸; Woodward *et al.*, 2010 ▸; Zarrella *et al.*, 2020 ▸). They possess CdaA as the sole DAC and the last three mentioned belong to the 12 bacterial species that pose the greatest threat to human health according to the WHO (Asokan *et al.*, 2019 ▸). This renders CdaA a particularly intriguing target for broad-spectrum therapeutics, especially against multidrug-resistant bacteria, as DACs are absent in humans (Rosenberg *et al.*, 2015 ▸). The recently reported biochemical and structural details of *L. monocytogenes* (*Lm*) CdaA provide a solid foundation for the development of a potent CdaA inhibitor using fragment-based drug discovery (FBDD; Erlanson *et al.*, 2016 ▸; Wollenhaupt *et al.*, 2021 ▸), a modern technique that is playing an increasingly important role in the development of new clinical candidates. This method uses small-molecule probes or fragments, which generally possess weak binding affinities to their target (Giordanetto *et al.*, 2019 ▸), that allow a thorough search of the available chemical space and provide starting points for drug development. In combination with X-ray crystallography, this enables structure-based drug discovery from the outset and can lead to the development of superior molecules.

Here, we present details of the crystallographic fragment-screening campaign for the bacterial target *Lm* CdaA utilizing the previously commercially available Frag Xtal Screen (Huschmann *et al.*, 2016 ▸; Jena Bioscience, MiTeGen) and eight fragment crystal structures derived from it. The target protein crystallized in the ortho­rhombic system with a noncatalytic ‘back-to-back’ homodimer in the asymmetric unit. The two active sites (one per monomer) are exposed to solvent, which makes them accessible to small-molecule ligands and favors soaking experiments. Two of the identified fragments that have been localized in the ATP-binding site of CdaA could serve as potential starting points for antibiotic drug discovery and for ‘growing’ the computationally designed lead candidate comprising two substituted thiazole rings (compound 7; Neumann *et al.*, 2023 ▸). This *in silico*-designed compound showed inhibitory potential based on ITC measurements and is the first structurally characterized CdaA inhibitor to date.

## 2. Experimental procedures

### 2.1. Bacterial strains and growth conditions

*Escherichia coli* BL21 (DE3) cells, which were used for overexpression of the protein, were cultivated in 2×YT medium comprising 1.6%(*w*/*v*) tryptone, 1.0%(*w*/*v*) yeast extract and 0.5%(*w*/*v*) NaCl. The transformed cells were selected on lysogeny broth medium plates containing ampicillin (100 µg ml^−1^).

### 2.2. Protein expression and purification

Protein expression and purification were performed as described previously (Heidemann *et al.*, 2019 ▸; Neumann *et al.*, 2023 ▸). Briefly, the plasmid pGEXpBP33, originating from the pGEX-6P-1 (Cytiva) vector and encoding a truncated *Lm *CdaA protein (Δ100CdaA) with an N-terminal GST tag, was transformed into *E. coli* BL21 (DE3) cells. The resulting cell cultures were grown in 2×YT medium at 37°C. Protein expression was induced at an OD_600_ of ∼0.6 by the addition of 1 m*M* isopropyl β-d-1-thiogalactopyranoside and the cultures were incubated overnight at 16°C. The harvested cells were disrupted with a microfluidizer (M-110S Microfluidizer, Microfluidics) in 20 m*M* Tris–HCl pH 7.5, 10 m*M* EDTA, 1 *M* NaCl and then centrifuged for 30 min at 4°C. The retained lysate containing the GST-tagged target protein was loaded onto a Glutathione Sepharose column (Cytiva) and eluted with 40 m*M* reduced GSH. The tag was proteolytically cleaved with 1:100(*w*:*w*) PreScission protease overnight at 4°C in cellulose tubing placed in dialysis buffer (300 m*M* NaCl, 20 m*M* Tris–HCl pH 7.5). The remaining impurities and the tag were removed using a Superdex 75 column (Cytiva) coupled to a Glutathione Sepharose column in 20 m*M* Tris–HCl pH 7.5, 300 m*M* NaCl. The protein was concentrated to 20 mg ml^−1^.

### 2.3. Crystallization

The sitting-drop vapor-diffusion method was applied for crystallization. The previously reported crystallization condition for apo CdaA (Heidemann *et al.*, 2019 ▸) was optimized in order to yield crystals that were more suited for fragment screening, *i.e.* had better diffraction properties (Neumann *et al.*, 2023 ▸). Crystallization trials were performed at 20°C using a concentration of 6 mg ml^−1^ Δ100CdaA in 2 µl droplets and a 1:1 protein:reservoir ratio. In order to facilitate crystal growth, microseeding was performed. Thin crystal plates were obtained overnight in 3.7 *M* NaCl, 0.1 *M* Na HEPES pH 8.5, 3% DMSO. The ideal DMSO concentration had been determined by previous stability tests.

### 2.4. Fragment soaking, data collection and structure determination

The Frag Xtal Screen fragment-screening library (Jena Bioscience, MiTeGen), comprising 96 fragments, each present in a dried form in two lenses of the respective well of a 96-well crystallization plate, was used. Solubilization of the dried fragments was achieved by pipetting 0.5 µl of the crystallization reservoir into one of the two lenses, resulting in a nominal fragment concentration of 100 m*M*. The second lens was supplemented with cryoprotecting buffer (crystallization reservoir saturated with sucrose; Heidemann *et al.*, 2019 ▸). The crystallization plates were sealed and left overnight at 20°C. Subsequently, two to four apo CdaA crystals were transferred to each well with the solubilized fragment. All crystals were soaked overnight, captured in SPINE-standard cryo-loops and cryoprotected in the second drop prior to plunging them into liquid nitrogen.

**Figure 2:**
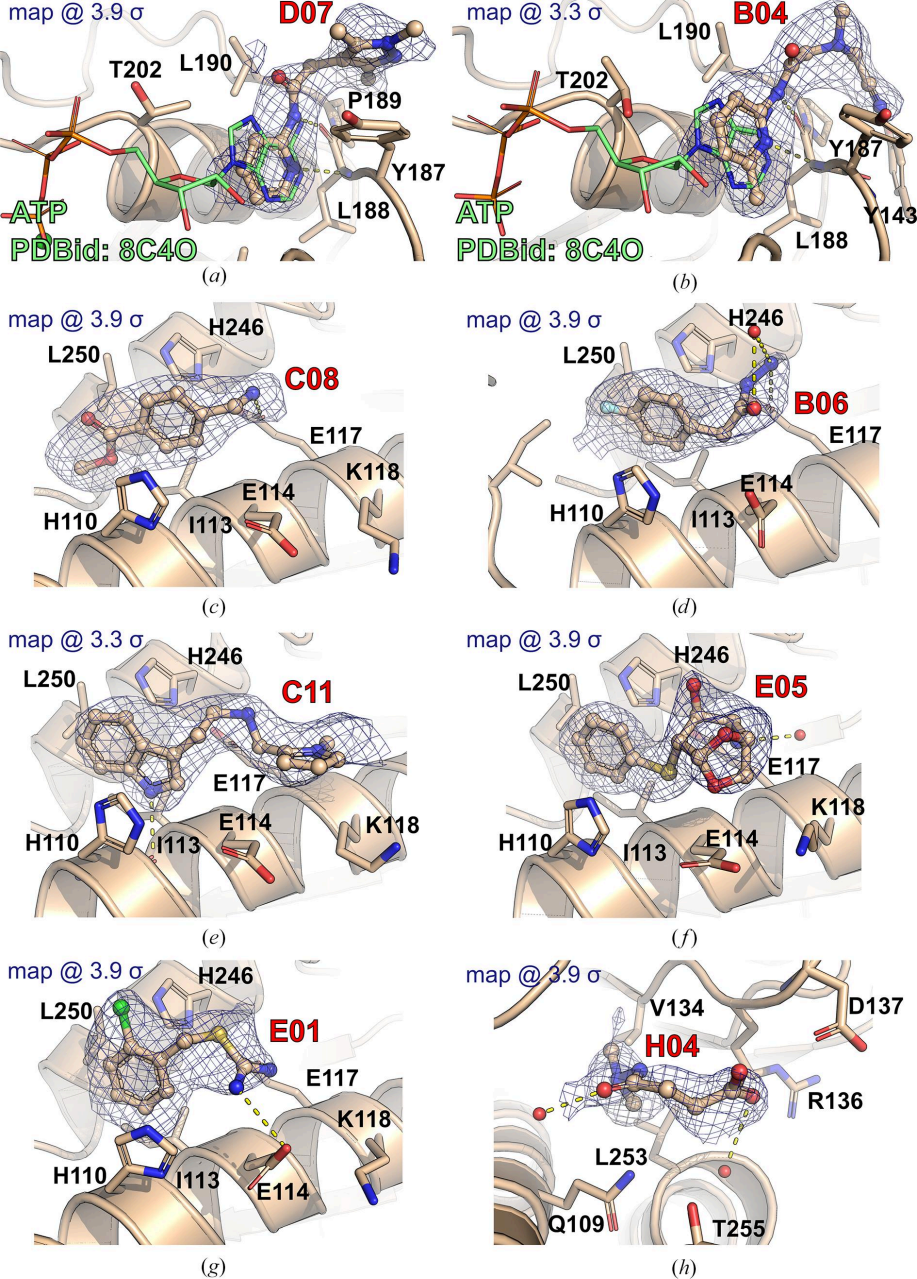
Detailed views of the binding sites of the eight identified fragments. Protein residues within 4 Å are depicted as sticks; hydrogen bonds are marked as yellow dashed lines. The polder omit map (*mF*_o_ − *DF*_c_) is shown at either the 3.3σ or the 3.9σ level. (*a*) D07, (*b*) B04, (*c*) C08, (*d*) B06, (*e*) C11, (*f*) E05, (*g*) E01, (*h*) H04. For fragments bound in the active sites (*a*) and (*b*), an ATP molecule is shown for reference (depicted as a green stick model, PDB entry 8c4o).

Diffraction images were collected on EMBL beamline P13 at PETRA III, DESY, Hamburg, Germany and the MASSIF-3 beamline at ESRF, Grenoble, France. All diffraction images were processed using a custom script (Neumann & Tittmann, 2014 ▸) utilizing the *XDS* package (Kabsch, 2010 ▸). Structure solution was performed using *DIMPLE* (Wojdyr *et al.*, 2013 ▸) employing programs from the *CCP*4 suite (Murshudov *et al.*, 2011 ▸; Agirre *et al.*, 2023 ▸). The resulting atomic models were refined using a customized self-written refinement pipeline making use of the *Phenix* package (*phenix.refine* and *phenix.real_space_refine*; Afonine *et al.*, 2018 ▸; Liebschner *et al.*, 2017 ▸, 2019 ▸). The refined structural models were subjected to *PanDDA* (Pearce *et al.*, 2017 ▸) to facilitate the identification of bound fragment molecules. Selected structures with well defined fragment molecules were manually inspected in *Coot* (Emsley *et al.*, 2010 ▸) and refined in *Phenix* using the default restrained refinement strategy (isotropic ADP refinement for all non-H atoms, automated TLS group assignment for protein molecules, optimization of the bulk-solvent mask and weights for stereochemical and ADP restraints). Ligand libraries were generated with *phenix.elbow*. Omit electron-density maps were calculated using *phenix.polder* (Liebschner *et al.*, 2017 ▸; Fig. 2 ▸). The identified small-molecule fragments are listed in Table 1 ▸. Data-collection, processing and refinement statistics are summarized in Tables 2 ▸ and 3 ▸.

### 2.5. Binding-site assignment of fragment molecules located between symmetry mates

**Figure 3:**
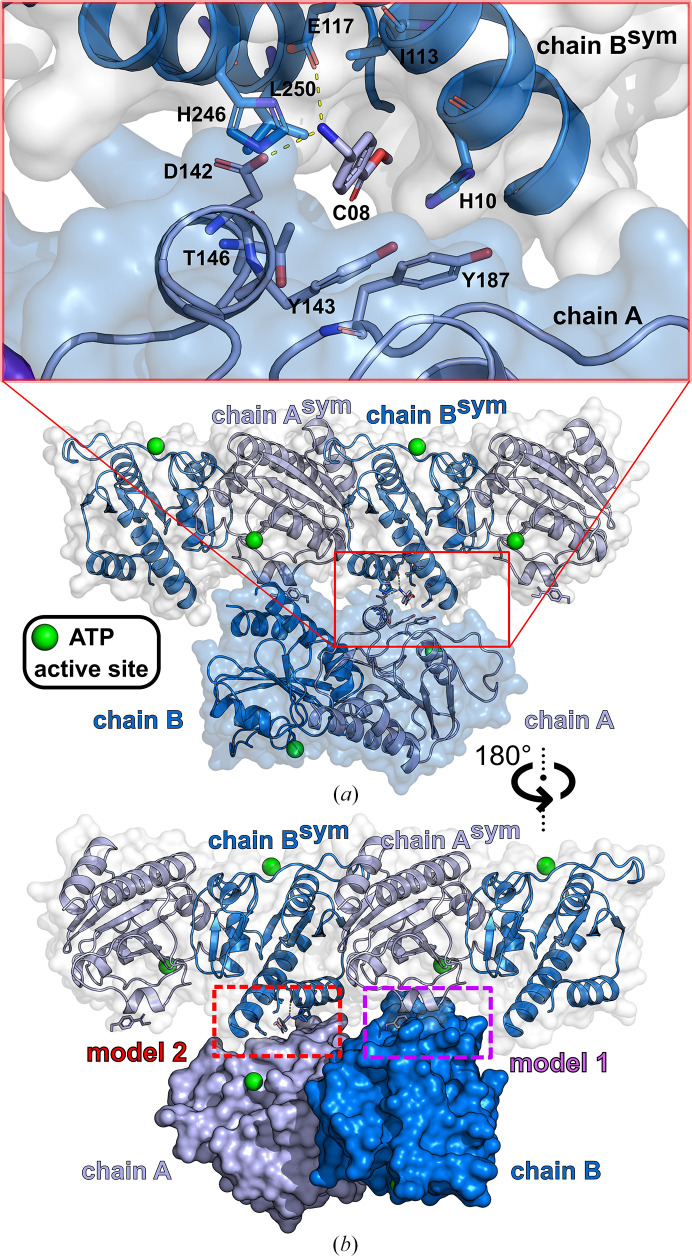
Binding-site assignment of fragment molecules located between symmetry-related molecules. (*a*) The asymmetric unit of the ortho­rhombic CdaA crystal (blue semi-transparent surface) with two neighboring symmetry-related dimers (cartoon representation, white semi-transparent surface). A close-up view shows a detailed representation of the fragment interactions (C08). (*b*) A 180° rotated view of (*a*). The alternative assignments of the C08 fragment molecule are depicted as model 1 (distant from the active site) and model 2 (closer proximity to the ATP-binding site).

The binding sites of five small-molecule fragments from the Frag Xtal Screen (B06, C08, C11, E01 and E05) were clearly localized between a CdaA molecule occupying the asymmetric unit (chain *A*) and a symmetry mate of the second CdaA molecule: chain *B* ^sym^ (Fig. 3 ▸). Initially, the inspected fragment molecules were modeled distant from the ATP-binding site and were therefore assigned to the CdaA molecule labeled as chain *B* (model 1, Fig. 3 ▸). The refined atomic models of these five CdaA complexes (model 1) were slightly manipulated to generate alternative structural models from the fragment perspective. In these generated models, the fragment molecules were placed in closer proximity to the ATP active site of the CdaA molecule labeled chain *A* (model 2), as shown in Fig. 3 ▸. Repositioning of the fragment molecules was performed in *Coot* using a selected symmetry mate as a reference. Subsequently, the two alternative structural models (models 1 and 2) were decomposed into two components: ligand (fragment) and receptor (protein). The receptor, which is identical for both models, was converted into PDBQT format using the *MGL* tools (Sanner, 1999 ▸). For each receptor–ligand pair, the ligand pose (the position of the fragment within the putative binding site) was reassessed using the molecular-docking program *Gnina* (McNutt *et al.*, 2021 ▸) utilizing an ensemble of convolutional neural networks (CNNs) as a scoring function. In addition, three frequently used scoring functions, *AutoDock* 4 (*AD*4), *Vina* and *Vinardo*, which provide a computationally predicted binding energy (affinity), were also tested. For this purpose, we used both *Vina* (version 1.2.5; Eberhardt *et al.*, 2021 ▸; Trott & Olson, 2010 ▸) and the *Gnina* program, the latter without CNN rescoring, thus simulating the *Vina* run. It should be noted that the commonly used scoring functions are likely to give non-identical results in terms of different predicted free energies of binding (predicted binding affinities) due to the way that they were developed: *Vinardo* is an empirical (regression-based) scoring function, *AutoDock* 4 is a physics-based scoring function and *Vina* is a hybrid scoring function. The final assignment of the CdaA molecule, with the estimated dominant contribution to the binding event, was based on the difference of the calculated CNN score and the lower predicted binding affinity, providing a comprehensive and robust basis for the selection of the most appropriate fragment-binding site. The proposed binding-site evaluation is computationally inexpensive (less then 5 s per fragment) and in addition it can easily be performed and scripted, as *Gnina* and *Vina* do not require any dictionaries or additional parameters for the fragment molecules.

## 3. Results

### 3.1. Identification of bound fragments

**Figure 4:**
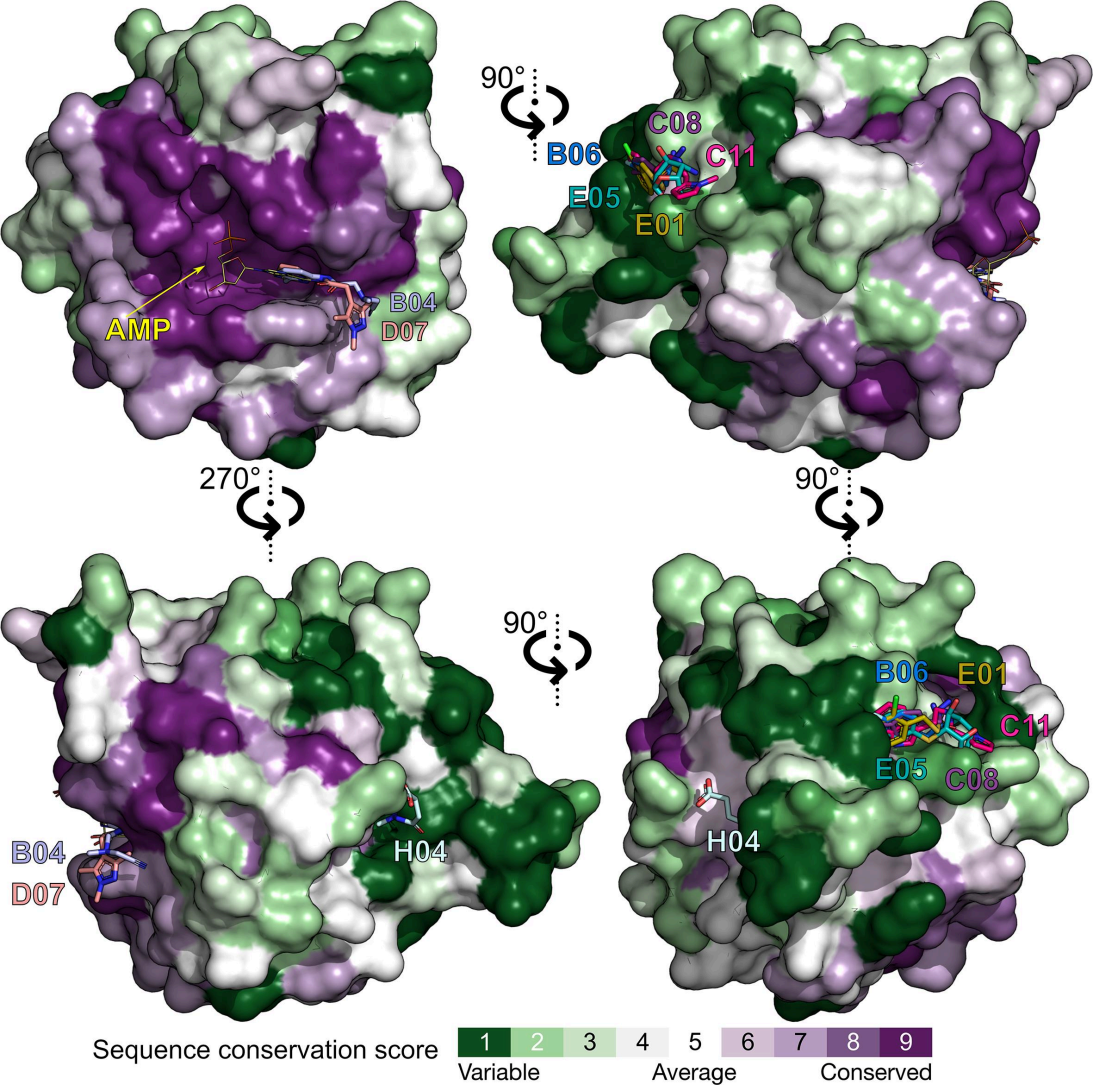
The eight fragment molecules (identified binding events) mapped on the surface of the CdaA monomer and colored according to the surface-conservation score. The individual views (90° increments) represent different views of the CdaA monomer. Two fragment molecules (B04 and D07) were localized in the highly conserved region (active site, marked with the AMP molecule colored yellow), while the remaining six fragments (B06, C08, C11, E01, E05 and H04) were identified in the nonconserved regions.

*L. monocytogenes* (*Lm*) CdaA is a membrane-bound protein consisting of an N-terminal transmembrane domain (residues 1–80), a 20-amino-acid flexible linker and a DAC domain (residues 101–255, abbreviated as Δ100CdaA; the numbering corresponds to that of *L. monocytogenes*). In the orthorhombic CdaA crystals, the asymmetric unit consists of two Δ100CdaA monomers forming a noncatalytic dimer with two outward-facing and solvent-accessible active sites. Therefore, it is expected that certain fragments can bind to both monomers. Using the Frag Xtal Screen, a 96-fragment library, fragment screening was performed and resulted in the collection of approximately 200 data sets (two to three crystals per fragment, together with some apo crystals). Remarkably, about 15% of the soaked crystals did not diffract or dissolved during the soaking experiments, while approximately 69% of all measured crystals diffracted to between 1.7 and 2.5 Å resolution. The macromolecular crystallography pipeline for refinement and ligand screening (*DIMPLE*) suggested only one structure with an unmodeled electron-density map (bound fragment molecule) as reported by the ‘Find Blob’ function using the default settings. Therefore, further analysis of all structures was performed with *PanDDA*. Manual inspection of the *DIMPLE*-based electron-density maps of the *PanDDA* hits revealed that all of them were placed in prominent blobs of the *mF*_o_ − *DF*_c_ electron-density map at the +3σ level that went undetected by *DIMPLE*. A total of eight fragments (Table 1 ▸ and Fig. 4 ▸) were identified. The polder omit maps for all fragments are shown in Fig. 2 ▸. Five fragments (B06, C08, C11, E01 and E05) were bound between one CdaA molecule (chain *A*) occupying the asymmetric unit and the symmetric counterpart of the second molecule (chain *B* ^sym^; Fig. 3 ▸). This led to ambiguity in assignment of the fragment-binding site to a single protein molecule (chain) within the crystal lattice. To resolve this ambiguity, the choice of binding-site assignment to a particular CdaA molecule was aided by a molecular docking-based approach using an ensemble of convolutional neural networks (CNNs) as well as predicted binding affinities to assess which protein molecule makes the largest contribution to the binding event. The binding sites of the remaining three fragments (C11, D07 and H04) could be unambiguously assigned, as the first two were located in the active site of CdaA and the last one was located on the solvent-exposed surface of a single CdaA molecule.

### 3.2. Molecular docking-based assessment of the binding-site localization

**Figure 5:**
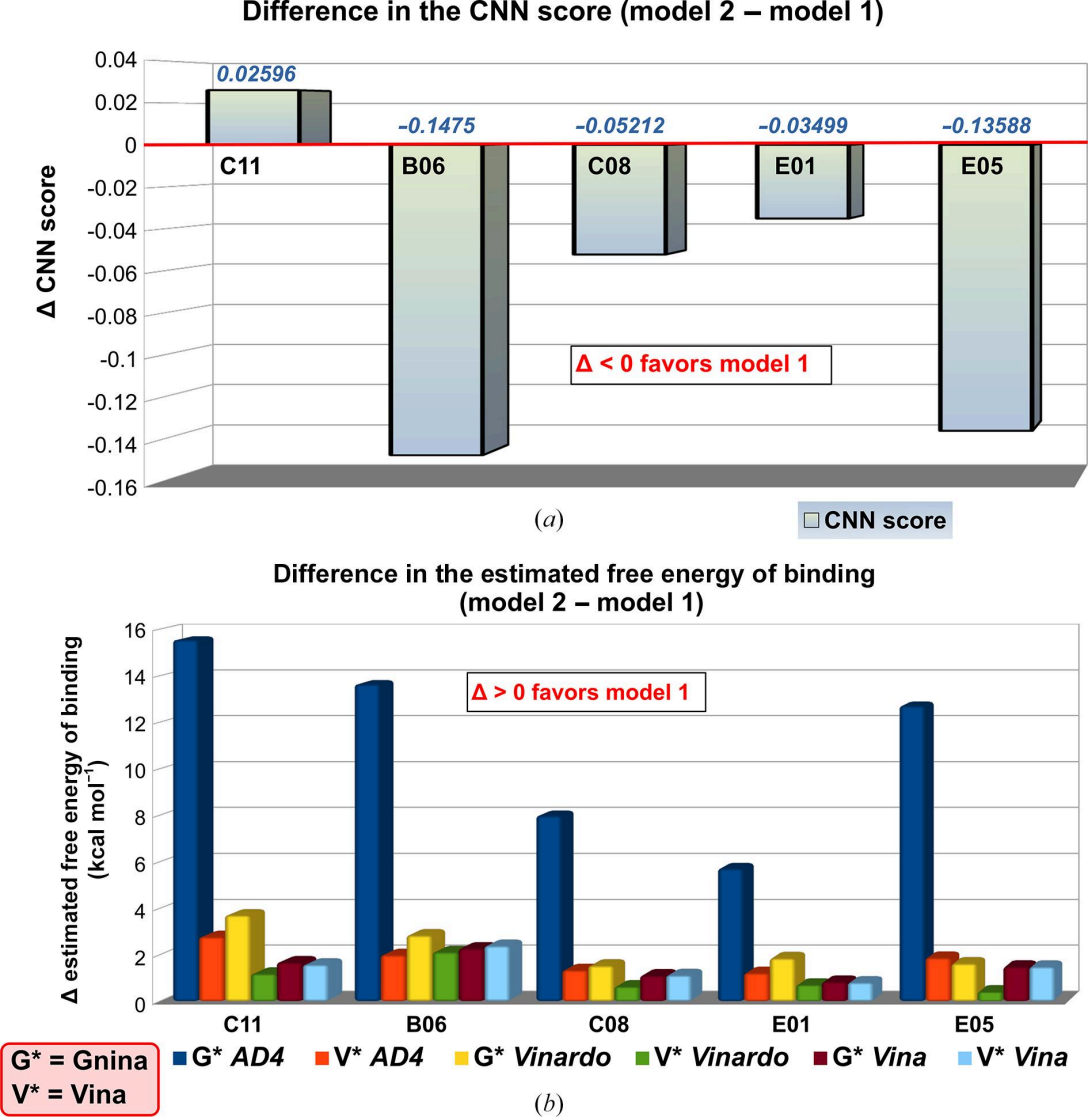
Molecular docking-based assessment of binding-site localization for five fragments located at the interface between symmetry-related CdaA molecules. (*a*) Differences between the *Gnina*-calculated CNN scores assessed for model 2 and model 1. The negative difference indicates that the calculated CNN score for model 1 is higher than that for model 2. (*b*) Differences in predicted binding affinity. Rescoring of fragment positions was calculated with the *Gnina* (G*) and *Vina* (V*) programs using three scoring functions: *AD*4, *Vinardo* and *Vina*. A positive difference indicates that the estimated free energy of binding (binding affinity) for model 1 is lower (more negative) than that for model 2 (less negative).

Molecular docking is a computational procedure that predicts the conformation of a small molecule (ligand) binding noncovalently to a receptor (protein). This prediction outputs both the conformation and the evaluated fitness of the pose, usually in the form of the binding affinity of the small molecule in its predicted minimum-energy state. These features have enabled the widespread use of this method for the virtual screening of large libraries of compounds. Recently, a new docking program, *Gnina* (McNutt *et al.*, 2021 ▸), that uses a CNN scoring function to re-evaluate the output poses has been released and made available to the scientific community. It outperforms other docking programs on redocking and cross-docking tasks when the binding pocket is defined (McNutt *et al.*, 2021 ▸). This inspired us to use *Gnina* to assess the fitness of the crystallographically refined positions of five fragment molecules (B06, C08, C11, E01 and E05) identified based on prominent spots of the *mF*_o_ − *DF*_c_ electron-density map at the +3σ level. These fragments were located between two symmetry-related CdaA (receptor) molecules within the orthorhombic crystal lattice. Consequently, these fragment molecules can be formally assigned to two alternative positions on the CdaA surface, as shown in Fig. 3 ▸: close to the ATP active site (potentially significant for drug development; model 2) or far away from it (probably of lesser importance; model 1). Our primary objective was to evaluate and compare the fitness of these two alternative assignments (model 1 versus model 2) by simulating the idealized scenario in which the ligand is bound exclusively by one of the two CdaA molecules (no influence of the crystal lattice). We calculated the convolutional neural network (CNN) scores with *Gnina* and predicted binding affinities (*Gnina* and *Vina*; *AD*4, *Vina* and *Vinardo* scoring functions) for both ligand positions (model 1 and model 2) using the same receptor molecule (two CdaA monomers occupying the asymmetric unit), and compared the results (Fig. 5 ▸). The results obtained clearly show that in the case of four ligands (B06, C08, E01 and E05) our original assignment (model 1) was correct considering only the CNN scoring. In the case of fragment C11, the difference in the CNN score of 0.026 indicates a slightly larger contribution of the binding event originating from the region close to the ATP-binding site. These results are in very good agreement with the assessed differences in predicted binding affinity calculated using three scoring functions with the *Gnina* and *Vina* programs (Fig. 5 ▸). The affinity-based assessment clearly shows that the initial binding-site assignment (model 1) was correct for all five ligands studied, regardless of the program and scoring function used. Remarkably, both tested programs provided different calculated affinities when the *Vinardo* and *AD*4 scoring functions were employed. This is most likely due to different default values of weights applied to certain energetic terms, for example repulsion, hydrogen bonding, hydrophobic interactions and van der Waals, that are used by both tested programs. Despite the formal assignment of the fragment molecules to a specific CdaA molecule (chain *A* or *B*), the observed ΔCNN scores and computed binding affinities (Fig. 5 ▸) indicate a non-negligible contribution of both involved CdaA molecules to the binding of inspected fragments. This could imply that the probability of observing these binding events in other crystal forms or in solution might be relatively low.

### 3.3. Prospects for fragment-based drug development for *Lm* CdaA

**Figure 6:**
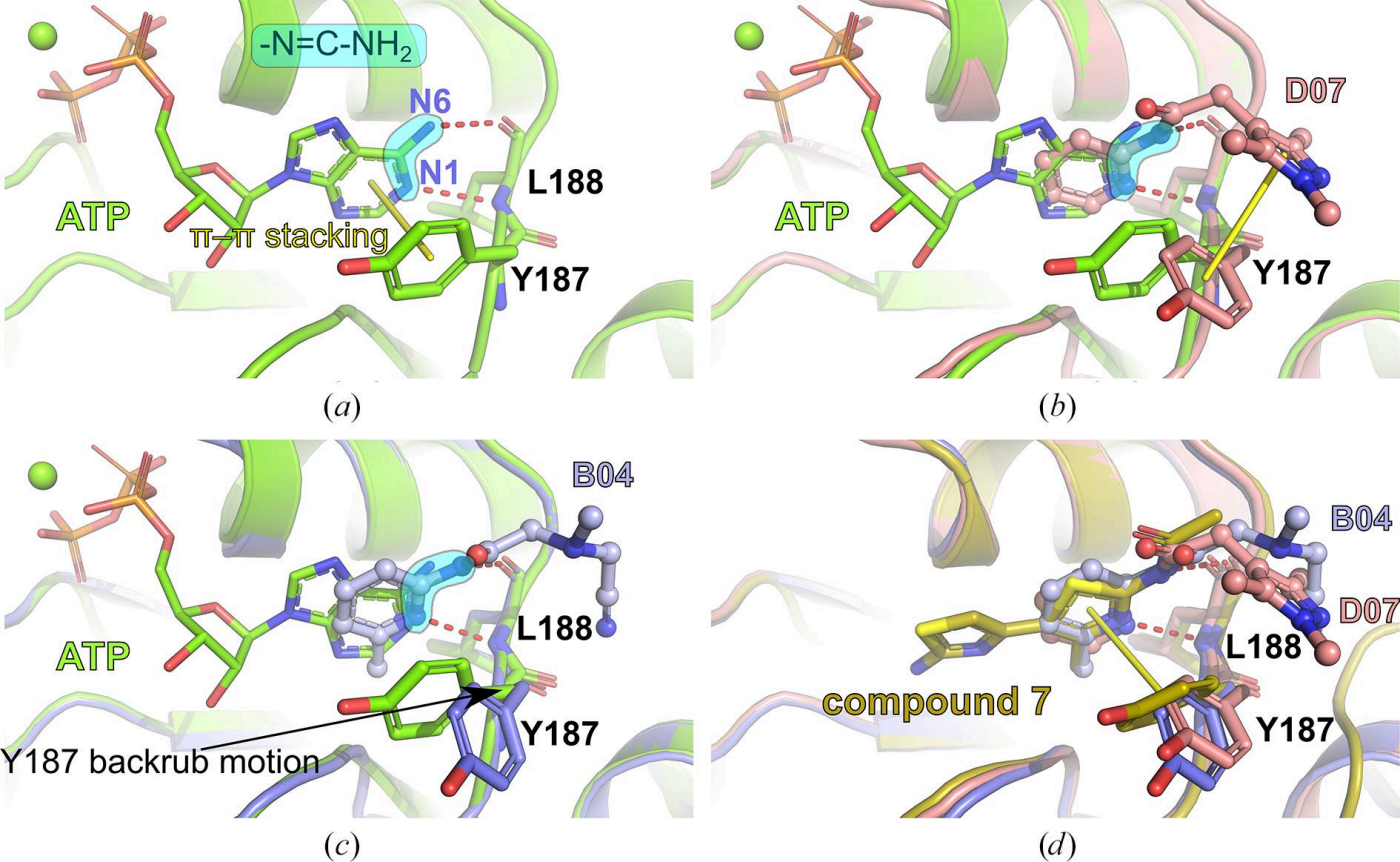
Comparison of the ATP-binding mode with fragments B04 and D07 and the *in silico*-designed CdaA inhibitor compound 7. The structural —N=C—NH_2_ pattern with two N atoms responsible for hydrogen-bond formation with the main-chain atoms of Leu188 is marked. The π–π stacking interactions are shown as yellow sticks. Tyr187 and Leu188 are shown as sticks. (*a*) ATP-binding mode in the active site of CdaA. (*b*) Superposition of CdaA structures with bound D07 and ATP molecules. (*c*) Superposition of the CdaA structures with bound B04 and ATP molecules. (*d*) Superposition of the structure of compound 7 with the structures of B04 and D07 provides a solid background for the development (‘growing’) of compound 7.

Using an assessment approach based on molecular docking, we aimed to achieve the unbiased assignment of a single protein molecule within the crystal lattice that predominantly contributes to the binding event. This was a prerequisite for evaluating the usability of the identified fragment molecules for the future development of a lead compound that will potentially inhibit several of the bacterial species that pose a major global health threat: in particular, those species that possess CdaA as the only DAC and constitute the group of 12 bacteria that pose the greatest threat to human health according to the WHO. Therefore, we mapped the eight identified binding events on the surface of the CdaA monomer colored according to the surface-conservation score (Fig. 4 ▸). Six of the eight localized fragments (B06, C08, C11, E01, E05 and H04) were identified in nonconserved regions on the CdaA surface. Moreover, the first five of these were located at nonbiologically relevant interfaces that are formed between two CdaA monomers and result from crystal packing. This unfavored positioning makes them unsuitable for the design of a general lead compound that should target several bacterial species that possess CdaA as the only enzyme synthesizing c-di-AMP. In contrast, only two fragment molecules (B04 and D07) localized in the highly sequence-conserved region (active site) are promising candidates for drug design and for further development (‘growing’) of the recently published CdaA inhibitor (Neumann *et al.*, 2023 ▸). These two fragments (B04 and D07) share the *N*-(2-pyridyl) moiety (Table 1 ▸), possessing the structural pattern —N=C—NH_2_, which has two adjacent N atoms that are structurally similar to N1 and N6 of the adenine ring (Figs. 2 ▸ and 6 ▸). This chemical and structural similarity leads to the formation of two hydrogen bonds similar to those observed for the adenine moiety (adenine N1–Leu188 N and adenine N6–Leu188 O). These two conserved hydrogen bonds have been observed in several CdaA complex structures (ADP, ATP and c-di-AMP), regardless of the postcatalytic or precatalytic state, the well described Tyr187 backrub motion and its π–π stacking interactions with aromatic rings of bound ligands (Fig. 6 ▸; Neumann *et al.*, 2023 ▸). Superposition of ATP-bound CdaA (PDB entry 8c4o) with the structure containing fragment D07 (Figs. 2 ▸ and 6 ▸) clearly shows the structural and positional equivalence of the —N=C—NH_2_ pattern. Its importance and its relevance for binding is further confirmed by fragment B04 (Figs. 2 ▸ and 6 ▸), which shows that even the presence of a hindering methyl group at position 6 [the *N*-(6-methyl-2-pyridyl) moiety] cannot prevent the formation of these two conserved hydrogen bonds. Remarkably, the aforementioned structural pattern was also used as a strong donor and acceptor constraint for the development of the bi-thiazole inhibitor (compound 7; Fig. 6 ▸), which binds to CdaA in the micromolar range and with a *K*_d_ ∼8-fold lower than that of the natural substrate ATP (Neumann *et al.*, 2023 ▸). Superposition of the crystal structure of the CdaA–compound 7 complex (PDB entry 8c4p) with those of the B04 and D07 fragment complexes reported here (Fig. 6 ▸) provides a solid background for the further development (‘growing’) of compound 7. This common strategy starting from prepositioned fragments and molecules has already been successfully used in structure-based design to identify high-affinity inhibitors for a variety of targets (Kick *et al.*, 1997 ▸; Liebeschuetz *et al.*, 2002 ▸).

## 4. Discussion

To facilitate the development of antibacterial therapeutics targeting *Lm* CdaA, we recently determined several CdaA crystal structures in apo (1.45 Å resolution), substrate ATP-bound (1.97 Å resolution; Neumann *et al.*, 2023 ▸) and post-catalytic c-di-AMP-bound states (Heidemann *et al.*, 2019 ▸), as well as a complex structure with the *in silico*-designed CdaA inhibitor (compound 7) at 1.2 Å resolution (Neumann *et al.*, 2023 ▸). These structures provide insight into the metal-dependent catalytic mechanism and confirmed the druggability of the highly conserved active site (Figs. 1 ▸ and 4 ▸). In order to explore the possibility of identifying additional binding pockets and to probe the available chemical space, a crystallographic fragment screen with 96 fragments was performed. Our main goal was to explore the possibility of obtaining CdaA complexes with non-ATP-like molecules that can be used for structure-guided optimization and the design of new compounds, with the future prospect of the rational design of antimicrobial therapeutics. We opted for soaking experiments, even though the crystallization conditions were not optimal (the buffer contained 3.6 *M* NaCl) and probably affected the solubilization of several of the compounds tested. Consequently, the hit rate of the fragment-screening campaign (a fragment binding to a particular site of the protein) was relatively low (∼8%). Nevertheless, despite the high-salt soaking conditions, the localized fragment molecules showed high occupancies and strong and well defined electron-density maps (Fig. 2 ▸). The two fragments identified in the conserved active site of CdaA, which share the *N*-(2-pyridyl) moiety, underline the importance of the —N=C—NH_2_ structural pattern known from the adenine ring, which seems to be crucial for binding. This chemical and structural constraint could be beneficial for the future development of an antibacterial therapeutic for several bacteria that should target CdaA from different species, for example *Listeria monocytogenes*, *Borrelia turicatea*, *Entero­coccus faecalis*, *Staphylococcus aureus*, *Mycobacterium tuberculosis* and *Streptococcus pneumoniae*. The first step towards this goal is the optimization of the abovementioned *in silico*-designed CdaA inhibitor (compound 7) with the fragment molecules (B04 and D07) reported here, which share the *N*-(2-pyridyl) moiety. The optimization should be accompanied by the structural and biochemical characterization of CdaA from other bacterial species, preferably multidrug-resistant *E. faecalis* and *S. aureus*. Taken together, this would be a small step towards an important goal: the expansion of our effective antimicrobial arsenal.

In recent years, especially since the beginning of the COVID-19 pandemic, several crystallographic fragment-screening campaigns have been conducted and made available to the scientific community to facilitate the development of new antiviral therapeutics (Boby *et al.*, 2023 ▸). X-ray crystallography is an excellent technique that provides structural information that enables the rapid and efficient assessment of hits that can subsequently be used for fragment-based drug discovery. However, the binding events must be carefully evaluated to account for artifacts such as fragment binding between symmetry-related protein interfaces. Therefore, the influence of the crystal lattice on fragment binding should not be neglected in both co-crystallization and soaking experiments. In some cases, when the target macromolecule forms oligomers that are either present in the asymmetric unit or formed by the crystallographic symmetry, the contribution of the lattice is beneficial. In many other cases, however, the binding site shared by the molecules indicates that the binding event is a product of the contribution of more than one protein molecule. It is beyond the scope of this manuscript to discuss the utility of such binding events for fragment-based drug design. Regardless of this, these events are also probes of the protein surface and could therefore provide useful insights into preferred chemical groups, undiscovered side-chain flexibility *etc*. Therefore, evaluating which receptor molecule is the major contributor to the observed binding event is crucial for successful fragment-based drug design relying on the most reliable fragment-binding region as a starting point.

## Supplementary Material

PDB reference: CdaA, complex with fragment E05, 8s45

PDB reference: complex with fragment E01, 8s46

PDB reference: complex with fragment D07, 8s47

PDB reference: complex with fragment C11, 8s48

PDB reference: complex with fragment B06, 8s49

PDB reference: complex with fragment B04, 8s4a

PDB reference: complex with fragment C08, 8s4b

PDB reference: complex with fragment H04, 8s4c

## Figures and Tables

**Table 1:** Fragment molecules

Plate ID	Fragment	SciFinder name	CAS ID	MW (g mol^−1^)
B04	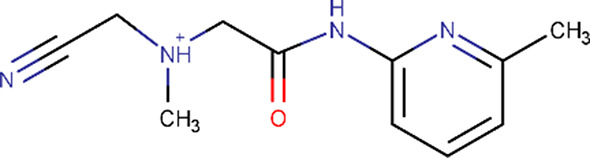	Acetamide, 2-[(cyanomethyl)methylamino]-*N*-(6-methyl-2-pyridinyl)-	1311649-76-9	218.25
B06	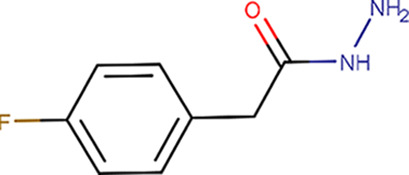	2-(4-Fluorophenyl)acetohydrazide	34547-28-9	168.17
C08	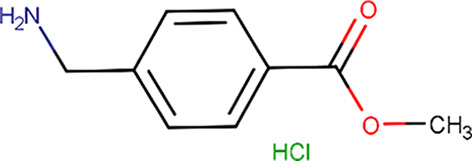	Methyl 4-(aminomethyl)benzoate hydrochloride (1:1)	6232-11-7	201.65
C11	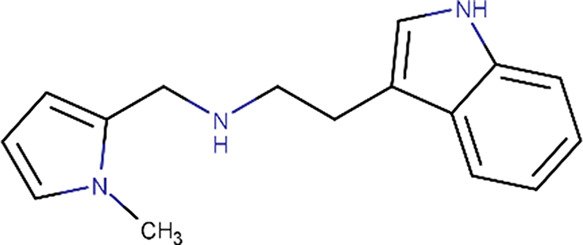	1H-Indole-3-ethanamine, *N*-[(1-methyl-1H-pyrrol-2-yl)methyl]-	289487-79-2	253.34
D07	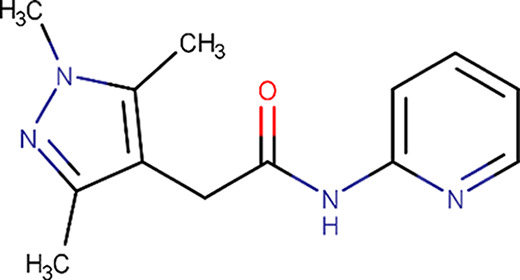	1H-Pyrazole-4-acetamide, 1,3,5-trimethyl-*N*-2-pyridinyl-	1171575-61-3	244.29
E01	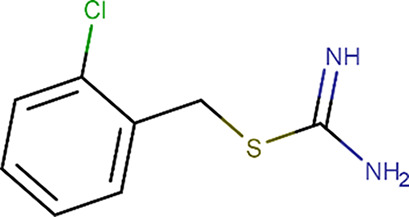	(2-Chlorophenyl)methyl carbamimidothioate	14122-38-4	200.69
E05	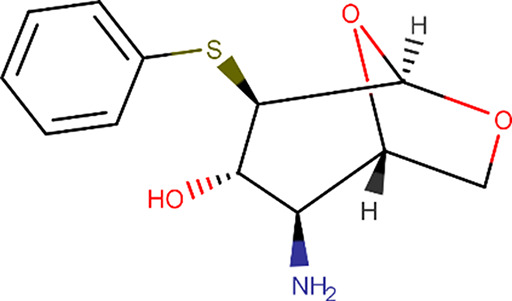	(1*S*,2*S*,3*S*,4*R*,5*R*)-2-Amino-4-phenylsulfanyl-6,8-dioxabicyclo[3.2.1]octan-3-ol	1212574-75-8	253.32
H04	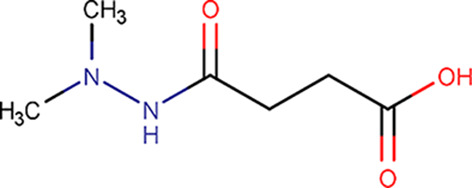	Butanedioic acid, 1-(2,2-dimethylhydrazide)	1596-84-5	160.17

**Table 2:** Data-collection statistics Values in parentheses are for the highest resolution shell.

Structure	D07	E01	E05	B06	B04	H04	C11	C08
PDB code	8s47	8s46	8s45	8s49	8s4a	8s4c	8s48	8s4b
Beamline	P13, PETRA III	MASSIF-3, ESRF	P13, PETRA III	MASSIF-3, ESRF	MASSIF-3, ESRF	MASSIF-3, ESRF	P13, PETRA III	P13, PETRA III
Wavelength (Å)	0.97625	0.96770	0.97625	0.96770	0.96770	0.96770	0.97625	0.97625
Space group	*P*2_1_2_1_2_1_	*P*2_1_2_1_2_1_	*P*2_1_2_1_2_1_	*P*2_1_2_1_2_1_	*P*2_1_2_1_2_1_	*P*2_1_2_1_2_1_	*P*2_1_2_1_2_1_	*P*2_1_2_1_2_1_
*a*, *b*, *c* (Å)	44.22, 65.07, 130.33	39.79, 64.65, 130.00	41.75, 64.71, 129.04	41.66, 64.75, 129.06	41.70, 64.46, 128.69	43.74, 64.11, 129.79	41.33, 64.68, 128.65	41.49, 64.72, 128.39
α, β, γ (°)	90, 90, 90	90, 90, 90	90, 90, 90	90, 90, 90	90, 90, 90	90, 90, 90	90, 90, 90	90, 90, 90
Resolution range (Å)	46.04–2.11 (2.16–2.11)	45.84–1.99 (2.03–1.99)	39.72–1.69 (1.71–1.69)	45.71–1.70 (1.72–1.70)	45.54–2.23 (2.30–2.23)	45.61–1.80 (1.83–1.80)	35.74–1.65 (1.67–1.65)	45.58–2.15 (2.25–2.15)
Total reflections	295034	194674	330217	311980	123142	288916	337480	149891
Unique reflections	41687 (2736)	44268 (2726)	74809 (2708)	73647 (2829)	31404 (2885)	65236 (2875)	79766 (2847)	36215 (4603)
Multiplicity	7.1	4.4	4.4	4.2	3.9	4.4	4.2	4.1
Completeness (%)	99.76 (99.45)	99.64 (99.67)	98.91 (97.10)	99.24 (98.64)	96.06 (95.72)	99.81 (99.62)	99.51 (98.44)	99.80 (99.08)
Mean *I*/σ(*I*)	10.08 (0.92)	11.58 (0.71)	13.10 (0.80)	10.06 (0.98)	8.04 (0.98)	13.12 (0.85)	10.42 (0.80)	5.68 (1.26)
Wilson *B* factor (Å^2^)	47.56	42.80	31.64	34.57	39.00	37.16	31.89	46.01
*R*_merge_ (%)	10.8 (265.1)	7.6 (258.9)	5.0 (205.5)	5.5 (204.6)	12.9 (148.6)	5.0 (173.9)	5.5 (180.8)	14.9 (136.5)
*R*_meas_ † (%)	11.7 (286.1)	8.6 (293.3)	5.7 (235.0)	6.4 (232.9)	14.9 (174.1)	5.7 (197.1)	6.3 (206.3)	17.1 (157.3)
CC_1/2_	99.9 (50.0)	99.9 (40.1)	99.9 (52.1)	99.8 (53.6)	99.6 (37.0)	99.8 (50.4)	99.9 (55.8)	99.3 (62.0)

† *R*_meas_ is the redundancy-independent multiplicity-weighted *R* factor for comparing symmetry-related reflections (Diederichs & Karplus, 1997 ▸).

**Table 3:** Crystallographic refinement statistics Values in parentheses are for the highest resolution shell.

Structure	D07	E01	E05	B06	B04	H04	C11	C08
PDB code	8s47	8s46	8s45	8s49	8s4a	8s4c	8s48	8s4b
Resolution range (Å)	46.04–2.11	45.84–1.99	39.72–1.69	45.71–1.70	45.54–2.23	45.61–1.80	35.74–1.65	45.58–2.15
Reflections used, refinement	41687 (2736)	44268 (2726)	74809 (2708)	73647 (2829)	31404 (2885)	65236 (2875)	79766 (2847)	36122 (2755)
Reflections used for *R*_free_	2083 (139)	2219 (146)	3748 (137)	3698 (142)	1572 (142)	3278 (147)	3989 (145)	1801 (126)
*R*_work_	0.2059 (0.4197)	0.2247 (0.4747)	0.1964 (0.5552)	0.1974 (0.5124)	0.2008 (0.3377)	0.2015 (0.4860)	0.1908 (0.5033)	0.2400 (0.4334)
*R*_free_	0.2402 (0.5195)	0.2587 (0.4941)	0.2357 (0.6358)	0.2207 (0.4554)	0.2436 (0.3930)	0.2268 (0.5125)	0.2187 (0.5334)	0.2774 (0.4832)
No. of non-H atoms								
Total	2495	2418	2604	2532	2485	2576	2674	2415
Macromolecules	2366	2344	2416	2383	2332	2414	2428	2326
Ligands	38	13	17	14	33	11	19	12
Solvent	91	61	171	135	120	151	227	77
Protein residues	308	305	310	308	304	314	311	302
R.m.s.d., bond lengths (Å)	0.002	0.002	0.010	0.009	0.003	0.004	0.008	0.002
R.m.s.d., angles (°)	0.42	0.49	0.93	0.97	0.54	0.70	0.83	0.42
Ramachandran favored (%)	98.68	99.34	99.35	98.68	97.67	99.35	98.37	96.64
Ramachandran allowed (%)	1.32	0.66	0.65	1.32	2.33	0.65	1.63	3.36
Ramachandran outliers (%)	0.00	0.00	0.00	0.00	0.00	0.00	0.00	0.00
Rotamer outliers (%)	2.65	1.14	1.11	1.12	0.00	0.37	1.84	2.30
Clashscore	1.65	2.52	2.45	3.10	2.31	1.22	2.84	1.06
Average *B* factor (Å^2^)								
Overall	62.68	60.48	43.22	47.51	50.42	54.82	44.81	53.12
Macromolecules	62.40	60.57	43.07	47.22	50.21	54.48	44.45	53.31
Ligands	77.62	65.78	41.50	53.99	64.16	80.38	48.22	45.93
Solvent	63.74	56.01	45.50	51.97	50.88	58.50	48.36	48.49

## References

[bb1] Afonine, P. V., Poon, B. K., Read, R. J., Sobolev, O. V., Terwilliger, T. C., Urzhumtsev, A. & Adams, P. D. (2018). *Acta Cryst.* D**74**, 531–544. 10.1107/S2059798318006551 PMC6096492 29872004

[bb37] Agirre, J., Atanasova, M., Bagdonas, H., Ballard, C. B., Baslé, A., Beilsten-Edmands, J., Borges, R. J., Brown, D. G., Burgos-Mármol, J. J., Berrisford, J. M., Bond, P. S., Caballero, I., Catapano, L., Chojnowski, G., Cook, A. G., Cowtan, K. D., Croll, T. I., Debreczeni, J. É., Devenish, N. E., Dodson, E. J., Drevon, T. R., Emsley, P., Evans, G., Evans, P. R., Fando, M., Foadi, J., Fuentes-Montero, L., Garman, E. F., Gerstel, M., Gildea, R. J., Hatti, K., Hekkelman, M. L., Heuser, P., Hoh, S. W., Hough, M. A., Jenkins, H. T., Jiménez, E., Joosten, R. P., Keegan, R. M., Keep, N., Krissinel, E. B., Kolenko, P., Kovalevskiy, O., Lamzin, V. S., Lawson, D. M., Lebedev, A. A., Leslie, A. G. W., Lohkamp, B., Long, F., Malý, M., McCoy, A. J., McNicholas, S. J., Medina, A., Millán, C., Murray, J. W., Murshudov, G. N., Nicholls, R. A., Noble, M. E. M., Oeffner, R., Pannu, N. S., Parkhurst, J. M., Pearce, N., Pereira, J., Perrakis, A., Powell, H. R., Read, R. J., Rigden, D. J., Rochira, W., Sammito, M., Sánchez Rodríguez, F., Sheldrick, G. M., Shelley, K. L., Simkovic, F., Simpkin, A. J., Skubak, P., Sobolev, E., Steiner, R. A., Stevenson, K., Tews, I., Thomas, J. M. H., Thorn, A., Valls, J. T., Uski, V., Usón, I., Vagin, A., Velankar, S., Vollmar, M., Walden, H., Waterman, D., Wilson, K. S., Winn, M. D., Winter, G., Wojdyr, M. & Yamashita, K. (2023). *Acta Cryst.* D**79**, 449–461.

[bb2] Asokan, G. V., Ramadhan, T., Ahmed, E. & Sanad, H. (2019). *Oman Med. J.* **34**, 184–193. 10.5001/omj.2019.37 PMC6505350 31110624

[bb3] Bai, Y., Yang, J., Zhou, X., Ding, X., Eisele, L. E. & Bai, G. (2012). *PLoS One*, **7**, e35206. 10.1371/journal.pone.0035206 PMC3328451 22529992

[bb4] Blötz, C., Treffon, K., Kaever, V., Schwede, F., Hammer, E. & Stülke, J. (2017). *Front. Microbiol.* **8**, 1328. 10.3389/fmicb.2017.01328 PMC5508000 28751888

[bb5] Boby, M. L., Fearon, D., Ferla, M., Filep, M., Koekemoer, L., Robinson, M. C., Chodera, J. D., Lee, A. A., London, N., von Delft, A., von Delft, F., Achdout, H., Aimon, A., Alonzi, D. S., Arbon, R., Aschenbrenner, J. C., Balcomb, B. H., Bar-David, E., Barr, H. & Zvornicanin, S. N. (2023). *Science*, **382**, eabo7201.

[bb6] Cassini, A., Högberg, L. D., Plachouras, D., Quattrocchi, A., Hoxha, A., Simonsen, G. S., Colomb-Cotinat, M., Kretzschmar, M. E., Devleesschauwer, B., Cecchini, M., Ouakrim, D. A., Oliveira, T. C., Struelens, M. J., Suetens, C., Monnet, D. L., Strauss, R., Mertens, K., Struyf, T., Catry, B., Latour, K., Ivanov, I. N., Dobreva, E. G., Tambic Andraševic, A., Soprek, S., Budimir, A., Paphitou, N., Žemlicková, H., Schytte Olsen, S., Wolff Sönksen, U., Märtin, P., Ivanova, M., Lyytikäinen, O., Jalava, J., Coignard, B., Eckmanns, T., Abu Sin, M., Haller, S., Daikos, G. L., Gikas, A., Tsiodras, S., Kontopidou, F., Tóth, Á., Hajdu, Á., Guðlaugsson, Ó., Kristinsson, K. G., Murchan, S., Burns, K., Pezzotti, P., Gagliotti, C., Dumpis, U., Liuimiene, A., Perrin, M., Borg, M. A., de Greeff, S. C., Monen, J. C., Koek, M. B., Elstrøm, P., Zabicka, D., Deptula, A., Hryniewicz, W., Caniça, M., Nogueira, P. J., Fernandes, P. A., Manageiro, V., Popescu, G. A., Serban, R. I., Schréterová, E., Litvová, S., Štefkovicová, M., Kolman, J., Klavs, I., Korošec, A., Aracil, B., Asensio, A., Pérez-Vázquez, M., Billström, H., Larsson, S., Reilly, J. S., Johnson, A. & Hopkins, S. (2019). *Lancet Infect. Dis.* **19**, 56–66.

[bb7] Commichau, F. M., Heidemann, J. L., Ficner, R. & Stülke, J. (2019). *J. Bacteriol.* **201**, 1–14. 10.1128/JB.00462-18 PMC6287462 30224435

[bb8] Corrigan, R. M., Abbott, J. C., Burhenne, H., Kaever, V. & Gründling, A. (2011). *PLoS Pathog.* **7**, e1002217. 10.1371/journal.ppat.1002217 PMC3164647 21909268

[bb9] Corrigan, R. M. & Gründling, A. (2013). *Nat. Rev. Microbiol.* **11**, 513–524. 10.1038/nrmicro3069 23812326

[bb10] Diederichs, K. & Karplus, P. A. (1997). *Nat. Struct. Mol. Biol.* **4**, 269–275. 10.1038/nsb0497-269 9095194

[bb11] Eberhardt, J., Santos-Martins, D., Tillack, A. F. & Forli, S. (2021). *J. Chem. Inf. Model.* **61**, 3891–3898. 10.1021/acs.jcim.1c00203 PMC10683950 34278794

[bb12] Emsley, P., Lohkamp, B., Scott, W. G. & Cowtan, K. (2010). *Acta Cryst.* D**66**, 486–501. 10.1107/S0907444910007493 PMC2852313 20383002

[bb13] Erlanson, D. A., Fesik, S. W., Hubbard, R. E., Jahnke, W. & Jhoti, H. (2016). *Nat. Rev. Drug Discov.* **15**, 605–619. 10.1038/nrd.2016.109 27417849

[bb14] Giordanetto, F., Jin, C., Willmore, L., Feher, M. & Shaw, D. E. (2019). *J. Med. Chem.* **62**, 3381–3394. 10.1021/acs.jmedchem.8b01855 PMC6466478 30875465

[bb15] Gundlach, J., Rath, H., Herzberg, C., Mäder, U. & Stülke, J. (2016). *Front. Microbiol.* **7**, https://doi.org/10.3389/fmicb.2016.00804. 10.3389/fmicb.2016.00804 PMC4879592 27252699

[bb16] Heidemann, J. L., Neumann, P., Dickmanns, A. & Ficner, R. (2019). *J. Biol. Chem.* **294**, 10463–10470. 10.1074/jbc.RA119.009246 PMC6615682 31118276

[bb17] Huschmann, F. U., Linnik, J., Sparta, K., Ühlein, M., Wang, X., Metz, A., Schiebel, J., Heine, A., Klebe, G., Weiss, M. S. & Mueller, U. (2016). *Acta Cryst.* F**72**, 346–355. 10.1107/S2053230X16004623 PMC4854561 27139825

[bb18] Jackson-Litteken, C. D., Ratliff, C. T., Kneubehl, A. R., Siletti, C., Pack, L., Lan, R., Huynh, T. N., Lopez, J. E. & Blevins, J. S. (2021). *Infect. Immun.* **89**, e00787-20. 10.1128/IAI.00787-20 PMC8316131 33846120

[bb19] Kabsch, W. (2010). *Acta Cryst.* D**66**, 125–132. 10.1107/S0907444909047337 PMC2815665 20124692

[bb20] Kick, E. K., Roe, D. C., Skillman, A. G., Liu, G., Ewing, T. J. A., Sun, Y., Kuntz, I. D. & Ellman, J. A. (1997). *Chem. Biol.* **4**, 297–307. 10.1016/s1074-5521(97)90073-9 9195867

[bb21] Liebeschuetz, J. W., Jones, S. D., Morgan, P. J., Murray, C. W., Rimmer, A. D., Roscoe, J. M. E., Waszkowycz, B., Welsh, P. M., Wylie, W. A., Young, S. C., Martin, H., Mahler, J., Brady, L. & Wilkinson, K. (2002). *J. Med. Chem.* **45**, 1221–1232. 10.1021/jm010944e 11881991

[bb22] Liebschner, D., Afonine, P. V., Baker, M. L., Bunkóczi, G., Chen, V. B., Croll, T. I., Hintze, B., Hung, L.-W., Jain, S., McCoy, A. J., Moriarty, N. W., Oeffner, R. D., Poon, B. K., Prisant, M. G., Read, R. J., Richardson, J. S., Richardson, D. C., Sammito, M. D., Sobolev, O. V., Stockwell, D. H., Terwilliger, T. C., Urzhumtsev, A. G., Videau, L. L., Williams, C. J. & Adams, P. D. (2019). *Acta Cryst.* D**75**, 861–877. 10.1107/S2059798319011471 PMC6778852 31588918

[bb23] Liebschner, D., Afonine, P. V., Moriarty, N. W., Poon, B. K., Sobolev, O. V., Terwilliger, T. C. & Adams, P. D. (2017). *Acta Cryst.* D**73**, 148–157. 10.1107/S2059798316018210 PMC5297918 28177311

[bb24] McNutt, A. T., Francoeur, P., Aggarwal, R., Masuda, T., Meli, R., Ragoza, M., Sunseri, J. & Koes, D. R. (2021). *J. Cheminform*, **13**, 43. 10.1186/s13321-021-00522-2 PMC8191141 34108002

[bb25] Mehne, F. M. P., Gunka, K., Eilers, H., Herzberg, C., Kaever, V. & Stülke, J. (2013). *J. Biol. Chem.* **288**, 2004–2017. 10.1074/jbc.M112.395491 PMC3548507 23192352

[bb26] Murshudov, G. N., Skubák, P., Lebedev, A. A., Pannu, N. S., Steiner, R. A., Nicholls, R. A., Winn, M. D., Long, F. & Vagin, A. A. (2011). *Acta Cryst.* D**67**, 355–367. 10.1107/S0907444911001314 PMC3069751 21460454

[bb27] Neumann, P., Kloskowski, P. & Ficner, R. (2023). *microLife*, **4**, uqad021. 10.1093/femsml/uqad021 PMC10167629 37223749

[bb28] Neumann, P. & Tittmann, K. (2014). *Curr. Opin. Struct. Biol.* **29**, 122–133. 10.1016/j.sbi.2014.11.001 25460275

[bb29] Pearce, N. M., Krojer, T. & von Delft, F. (2017). *Acta Cryst.* D**73**, 256–266. 10.1107/S2059798317003412 PMC5349438 28291761

[bb30] Römling, U. (2008). *Sci. Signal.* **1**, pe39. 10.1126/scisignal.133pe39 18714086

[bb31] Rørvik, G. H., Liskiewicz, K. A., Kryuchkov, F., Naemi, A., Aasheim, H., Petersen, F. C., Küntziger, T. M. & Simm, R. (2020). *Microorganisms*, **8**, 1269. 10.3390/microorganisms8091269 PMC7570391 32825526

[bb32] Rosenberg, J., Dickmanns, A., Neumann, P., Gunka, K., Arens, J., Kaever, V., Stülke, J., Ficner, R. & Commichau, F. M. (2015). *J. Biol. Chem.* **290**, 6596–6606. 10.1074/jbc.M114.630418 PMC4358292 25605729

[bb33] Sanner, M. (1999). *J. Mol. Graph. Model.* **17**, 57–61. 10660911

[bb34] Schuster, C. F., Bellows, L. E., Tosi, T., Campeotto, I., Corrigan, R. M., Freemont, P. & Gründling, A. (2016). *Sci. Signal.* **9**, ra81. 10.1126/scisignal.aaf7279 PMC5248971 27531650

[bb35] Trott, O. & Olson, A. J. (2010). *J. Comput. Chem.* **31**, 455–461. 10.1002/jcc.21334 PMC3041641 19499576

[bb36] Whiteley, A. T., Garelis, N. E., Peterson, B. N., Choi, P. H., Tong, L., Woodward, J. J. & Portnoy, D. A. (2017). *Mol. Microbiol.* **104**, 212–233. 10.1111/mmi.13622 PMC5391996 28097715

[bb99] Witte, G., Hartung, S., Büttner, K. & Hopfner, K. (2008). *Mol. Cell*, **30**, 167–178. 10.1016/j.molcel.2008.02.020 18439896

[bb38] Wojdyr, M., Keegan, R., Winter, G. & Ashton, A. (2013). *Acta Cryst.* A**69**, s299.

[bb39] Wollenhaupt, J., Barthel, T., Lima, G. M. A., Metz, A., Wallacher, D., Jagudin, E., Huschmann, F. U., Hauss, T., Feiler, C. G., Gerlach, M., Hellmig, M., Förster, R., Steffien, M., Heine, A., Klebe, G., Mueller, U. & Weiss, M. S. (2021). *J. Vis. Exp.*, e62208. 10.3791/62208 33749678

[bb40] Woodward, J. J., Iavarone, A. T. & Portnoy, D. A. (2010). *Science*, **328**, 1703–1705. 10.1126/science.1189801 PMC3156580 20508090

[bb41] Zarrella, T. M., Yang, J., Metzger, D. W. & Bai, G. (2020). *J. Bacteriol.* **202**, https://doi.org/10.1128/jb.00691-19. 10.1128/JB.00691-19 PMC6989799 31767779

